# The Unmet Needs of Palliative Care Among Young and Middle-Aged Patients with Advanced Cancer: A Qualitative Study

**DOI:** 10.3390/curroncol32060314

**Published:** 2025-05-29

**Authors:** Renhui Wen, Xinyi Liu, Yu Luo

**Affiliations:** School of Nursing, Army Medical University (Third Military Medical University), Chongqing 400038, China; wenrenhui@tmmu.edu.cn (R.W.); xyliu7012@tmmu.edu.cn (X.L.)

**Keywords:** palliative care, care needs, cancer, young and middle-aged, China, qualitative study

## Abstract

Objective: This study aimed to explore the unmet palliative care needs among young and middle-aged (YMA) Chinese patients with advanced cancer. Methods: We used the principle of maximum difference. A total of 16 YMA patients with advanced cancer from cancer hospital were recruited. Semi-structured, in-depth, and face-to-face interviews were conducted from 28 August 2023 to 23 October 2023. The recorded audio of each interview was typed into Word software with each personal code. The interview transcripts were coded using the method of inductive content analysis. Results: Four themes and 14 sub-themes were identified in participants’ descriptions of care needs: (1) symptom management needs: need for pain relief, need for anti-emetics, and need for aid in managing fatigue; (2) psychological support needs: help reducing fear of pain, help achieving a better death, and help with parents’ negative reactions; (3) social support needs: taking care of children, emotional support from family members, consultation and emotional support from other cancer patients, and company and guidance of healthcare personnel; (4) information needs: better understanding of disease trajectory and future care needs, better access to palliative care information, and more participation in medical decision-making. Conclusions: According to the results of this study, the unmet palliative care needs of YMA patients with advanced cancer are diverse, but they have not been fully recognized and met. Therefore, medical staff should develop effective management strategies and explore patients’ needs in an all-around way. Future studies will further develop the scale of unmet needs for palliative care to accurately identify needs and improve patients’ quality of life.

## 1. Introduction

Global figures from the International Agency for Research on Cancer (IARC) indicated about 9.7 million deaths and 20 million new cases of cancer in the year 2022 [1], with new cases of cancer projected to exceed 35 million in 2050. A significant proportion of the global disease burden of cancer is concentrated in China. There were approximately 2.57 million deaths and 4.82 million new cases of cancer in China [2].

Palliative care is a comprehensive and active model of care that aims to manage and relieve pain while providing integrated physical, psychological, social, and spiritual care. It focuses on supporting people who are suffering from severe illnesses and experiencing significant pain [3]. Patients with malignant tumors, in particular, need such multi-dimensional support.

In China, the National Bureau of Statistics divides the age range of young and middle-aged people as 16 to 59 [4]. The incidence of cancer in Chinese young and middle-aged (YMA) is on the increase, especially cancer with a high degree of malignancy and rapid disease progression [2]. Moreover, YMA patients with cancer are the backbone of the family and society and often bear multiple responsibilities. Therefore, the impact of cancer fatigue and feelings of guilt towards the family are more apparent [5], while the pain experienced can be higher [6].

However, in China, influenced by traditional ideas and culture, the public’s acceptance of death and thus palliative care is relatively low. Moreover, most studies on palliative care focus on elderly patients, patients with metastasized tumors, and non-tumor chronic diseases [7]. Hence, little attention has been paid to the unmet needs for palliative care among YMA Chinese patients with advanced cancer.

Although it has been observed that the implementation of palliative care can reduce the unnecessary curative treatments (such as chemotherapy, radiotherapy, intubation resuscitation, etc.), reduce traumatic procedures, and reduce the financial burden, palliative care for YMA adults with advanced tumors are usually not involved or is involved late [8,9]. It is necessary to increase the palliative care participation rate of YMA patients with advanced cancer, understand the unmet needs of YMA patients with advanced cancer in receiving palliative care services, and improve the quality of life of patients. In previous studies, due to the bias against “palliative care”, the demand for middle-aged and young patients with advanced cancer mainly focused on support demand [10], nursing demand [11], and support preference [12]. However, supportive care services include palliative care, emergency oncology care, medical oncology care, surgical oncology care, oral care, allied health worker team support, and other services [13]. Palliative care is only one component of the supportive needs, which mainly include the physical, psychological, social, and spiritual needs of the patient. Therefore, this study focuses on the unmet needs of palliative care in YMA adults with advanced cancer to provide information for improving palliative care acceptance in this population.

Research Question: What are the unmet needs for palliative care in middle-aged and young adults with advanced cancer?

## 2. Materials and Methods

### 2.1. Study Design

This research employs a descriptive qualitative approach, describing and interpreting the experiences and actions of relevant individuals and groups in their social and cultural context. To understand the unmet palliative care needs of YMA patients with advanced cancer in Chongqing, China. The interviews were conducted by the author between 28 August 2023 and 23 October 2023 in a quiet room in the cancer hospital. One of the authors is a nurse working in a hospital and a graduate student, while the other is also a graduate student.

Ethics committee approval was obtained from the Chongqing University Cancer Hospital (CZLS2023230-A). The study was conducted in accordance with the principles laid down in the Declaration of Helsinki. All participants participated in the study voluntarily and signed written informed consent. Patients could withdraw from the study at any time. Interview data and recordings were kept confidential.

### 2.2. Setting and Participants

Participants were initially selected using maximum variation sampling to maximize the diversity of palliative care needs. According to gender, age, education level, cancer type, and diagnosis time, middle-aged and young patients with advanced cancer in a cancer hospital in Chongqing were selected. Analysis and data collection were carried out simultaneously; the preliminary analysis provided information for subsequent sampling to further recruit participants with characteristics that could extend existing topics. The researchers selected consenting participants who met the following inclusion criteria: (i) aged 18–59 years old; (ii) patients diagnosed with cancer, with a clinical TNM stage of III or IV; (iii) aware of their diagnosis of cancer; (iv) able to express and describe feelings well. Exclusion criteria were as follows: (i) patients with serious mental and cognitive abnormalities; (ii) patients in critical condition with serious complications of other systems. The number of participants was determined via the principle of data saturation. When the 16th patient was interviewed, the data analysis yielded no new topics. In order to verify whether the data were saturated, we interviewed two YMA cancer patients, and the obtained information completely overlapped with the data analysis results. Therefore, after discussion by the third researcher, the information in this study reached saturation [14].

### 2.3. Data Collection

The study data were collected through in-depth semi-structured face-to-face interviews. Research was undertaken by Renhui Wen and Xinyi Liu, both of whom have received systematic training in qualitative research and data analysis. The initial interview outline was formed by reviewing relevant literature and discussing with the research group, and the final interview outline was formed after pre-interviewing 2 advanced YMA cancer patients (Table 1). Before the interview, the researcher contacted the ward and, with the help of the ward manager, contacted the patients who met the inclusion criteria and were willing to participate. The researcher established a good relationship with the interviewees, informed them of the purpose and content of the study, and signed the informed consent after confirming that the patients agreed to participate in the study. In recording and processing the data, participants were anonymized (p1, p2, etc.).

### 2.4. Data Analysis

The collected data were analyzed using inductive content analysis, that is, content, code, categories, and topics were generated from text data [15]. Within 24 to 48 h after each interview, two researchers transcribed the interview audio recording verbatim into text and then coded the transcribed text. The analysis followed the method proposed by Graneheim and Lundman. (1) Each interview was read verbatim; (2) the interview content was divided into meaning units, which were summarized and simplified; (3) the meaning units were encoded and the encodings summarized; (4) encodings were compared for similarities, differences, and appropriateness, ensuring each code represented a single topic; (5) categories were then generated based on commonalities; (6) central concepts or sub-themes were identified and extracted and a concise and understandable theme generated for each category [16]. After coding, sub-themes and themes were reviewed to ensure they were valuable and accurate representations of the data. If opinions on coding differed, a third researcher was included in the process and a resolution reached via discussion.

### 2.5. Reliability

In order to ensure the scientific credibility of the research results, we improved the credibility, transferability, reliability and verifiability of the research from three aspects: researchers, research process, and research results analysis. Both researchers had received qualitative research system training at university and had mastered interview-related skills and precautions to ensure reliability. In the early stage, this study described the background of qualitative research, focusing on YMA patients with advanced cancer under the Chinese cultural background, clearly defined the inclusion and exclusion criteria, and used the principle of maximum differentiation to extract an appropriate sample size to ensure credibility. The interview outline is listed, the research process is reported in detail, and transferability was emphasized.

Two members of the research team independently extracted and coded themes from the interview content. When discrepancies in data analysis arose, group discussions were conducted to reach a consensus, and the corresponding author supervised the entire process to ensure verifiability.

## 3. Results

Overall, 16 YMA cancer patients of the target demographic were recruited and interviewed for this research. Each interview took approximately 30 min. The mean patient age was 45.44 ± 12.33 years. The demographic and disease information of each patient are included (Table 2). During qualitative analysis of unmet palliative care needs, four themes and fourteen sub-themes were identified in participants’ descriptions of care needs: (1) symptom management needs: need for pain relief, need for anti-emetics, and need for aid in managing fatigue; (2) psychological support needs: help reducing fear of pain, help achieving a better death, and help with parents’ negative reactions; (3) social support needs: taking care of children, emotional support from family members, consultation and emotional support from other cancer patients, and company and guidance of healthcare personnel; (4) information needs: better understanding of disease trajectory and future care needs, better access to palliative care information, and more participation in medical decision-making.

### 3.1. Symptom Management Needs

Need for pain relief: multiple patients expressed experiencing pain, and pain relief was one of the most urgent demands of patients.

“*Because with a disease like ours, it may be a kind of pain in the final stage, and the most important thing is to relieve our pain*”.

(P6)

Need for anti-emetics: patients undergoing chemotherapy drug infusion may experience nausea and vomiting, and this can be severe enough to cause anticipatory vomiting and psychological fear. Some indicated a desire for aid managing these symptoms.

“*During chemotherapy, I had no appetite and would vomit whenever I ate something. I lost dozens of pounds, and my nutrition couldn’t keep up either*”.

(P11)

Need for aid in managing fatigue: with the deepening of treatment, some cancer patients reported being limited in their activities by fatigue, forcing them to only rest in bed. Patients reported needing help to manage their fatigue.

“*Now I feel very tired even with just a little movement. I’m exhausted and still feel tired even after taking a rest*”.

(P16)

### 3.2. Psychological Support Needs

Help reducing fear of pain: patients reported finding the pain too torturous and fear-inducing. Desperate for relief, some patients turned to religion for help or had thoughts of death.

“*I’m not afraid of anything else but pain. So I pray to God that it would be okay if I could pass away suddenly and without suffering*”.

(P2)

Help achieving a better death: some patients reported desiring a better death, to not have to suffer too much before dying, and to leave with more dignity.

“*Only by living a life of quality can one pass away peacefully and without pain. I envy my father-in-law. He passed away suddenly, and that’s the best way to go*”.

(P2)

Help with parents’ negative reactions: some patients reported that family members felt that the hospital environment was too depressing and emotionally disturbing, and this made them reluctant to come to the hospital.

“*It’s quite necessary to give life and death education to family members. The main problem with them is that they can’t be open-minded. My mother doesn’t even want to come. She always says that the hospital is too depressing every time*”.

(P1)

### 3.3. Social Support Needs

Taking care of children: patients with advanced cancer are often accompanied by a series of symptoms such as fatigue, pain, nausea, vomiting, etc., resulting in a physical condition that is extremely inconsistent with the energy that should be required at this age and an inability to take good care of children.

“*My biggest concern is that I won’t be able to settle the children properly, and if I can settle them well, I won’t have any worries*”.

(sighs) (P12)

Emotional support from family members: some patients in the last stage of life reported a desire to feel the love of their family, especially those who indicated a desire to return to their hometowns.

“*For example, if I’m already at the end of my life, what I would probably prefer is to be with my family, to be with my grandma. I was brought up by my grandma since I was a child, and home is a warmer place*”.

(P4)

Consultation and emotional support from other cancer patients: some patients indicated that they desired to communicate and share with other cancer patients because of similar experiences.

“*Now, if my physical condition permits, I still want to chat with my fellow patients for a while every day, asking each other about what diseases we have and the course of our treatments*”.

(P10)

Company and guidance of healthcare personnel: some patients stated that they desired to communicate and interact more with their care providers.

“*We still hope that doctors and nurses can talk with us more, but they are too busy. It’s impossible for them to come and help you with psychological counseling individually*”.

(P4)

### 3.4. Information Needs

Better understanding of disease trajectory and future care needs: patients wanted doctors to tell them truthfully and objectively about the disease and how it would affect them.

“*Now with this kind of treatment, it seems that it can’t relieve the condition. I don’t know how long I can live.* (*The patient choked up*.)”

(P8)

Better access to palliative care information: after learning about palliative care, patients thought it a great idea but stated that it was the first time they had heard of it or that they had heard very little about it before.

“*I didn’t know about palliative care before. My aunt was undergoing rescue all the time back then. If I had known about palliative care, I would have chosen it. If I had understood palliative care, I think the decision I made at that time might have been different*”.

(P5)

More participation in medical decision-making: patients desired to make joint medical decisions with medical staff and be included in treatment steps.

“*I will take the initiative to look up information online, such as on CNKI. I will also communicate with fellow patients across the country on the Internet. Besides, I will make appointments with experts from other hospitals to learn about the disease and communicate with my attending doctor. Once I know that there is a standardized treatment plan for my disease, I can just follow the treatment step by step*”.

(P9)

## 4. Discussion

To the best of our knowledge, this is the first study to explore the palliative care needs among YMA patients with advanced cancer. Identification of unmet palliative care needs among YMA patients with advanced cancer and consideration of the complexity of these needs can help healthcare providers to provide them with the care services appropriate to their needs. Four themes with fourteen subcategories emerged from the codes taken from the interviews: symptom management needs, social support needs, psychological support needs, and information needs.

This study extracted the core theme of symptom management in the palliative care needs of YMA patients with advanced cancer. Effective management of pain, nausea, and fatigue constitutes the primary focus of these patients’ palliative care requirements. Our research is consistent with previous research demonstrating that preventing pain and other symptoms at the end of life is also a crucial component of quality palliative care for YMA cancer patients [17]. Among the 38 cancer-related symptoms, cancer-related fatigue, pain, and nausea and vomiting are the frequent complaints of patients with advanced cancer, with pain being the most common [18]. About two out of three patients with advanced cancer and those receiving palliative care report suffering from pain [19]. The high burden of symptoms associated with cancer is a huge challenge to patients and can have a substantial impact on cancer outcomes, treatment adherence, and quality of life [20,21]. The findings of the research by Chung et al. revealed that palliative care with an emphasis on aggressive symptom management may sustain both clinical and patient-centered outcomes throughout the course of treatment [22]. Findings from this study had a profound impact on the formulation of evidence-based pain and symptom management plans and the standardization of the collaborative approach of multidisciplinary teams for patients with terminal diseases.

One theme that emerged in this study centered on social support needs, including taking care of children, emotional support from family members, consultation and emotional support from other cancer patients, and company and guidance of healthcare personnel. Many participants in this study recognized that they are the backbone of the family and society and often bear multiple responsibilities. Previous studies have shown that 24.7% of cancer patients have children, and 22% of cancer patients have to bear the pressure of raising minor children while fighting the disease [23]. This highlights how cancer can have a devastating impact on YMA individuals and families [24]. Another qualitative study described how young-onset colorectal cancer patients reported feeling isolation, desiring social support, and experiencing life disruption due to the challenge of balancing many responsibilities [25]. The Social Cognitive Processing Model holds that people who possess more resources are less vulnerable to the harmful spiral of resource loss [26]. Social support is the moral or material support provided to an individual by various parties in the community and is a state resource possessed by the patient [27]. Findings from this study highlighted the multiple social supports (such as parenting, family emotional relief, peer palliative care need) [28].

Psychological support needs with the three subcategories were identified in the present research. YMA patients with advanced cancer experience complex emotional changes including psychological, social, and spiritual experiences, which can manifest as severe abnormal emotional responses such as depression, anxiety, fear, social isolation, and mental crisis [27]. Previous studies showed that approximately 30–50% of cancer patients exhibited multiple neuropsychological or mental symptoms [28]. Medical staff should provide for YMA cancer patients comfort care comprehensively with a focus on the patient’s body and mind. Studies have shown that without psychological intervention, the decline in quality of life caused by the above symptoms not only has immediate and short-term effects but also has long-term adverse effects up to 12 months after the end of the period [29]. Effective psychological intervention therapies are crucial for patients; therefore, on the basis of rational medication to alleviate patients’ psychological pain, medical providers in hospice wards may need to adopt cognitive-behavioral therapies, positive thinking therapies, respiratory training, and relaxation therapies, such as tai chi and yoga, to satisfy the unmet support needs of YMA cancer patients [30,31].

Information needs among YMA patients with advanced cancer emerged from this qualitative study. Recognizing and satisfying patients’ information needs is critical to improving the quality of their care, especially in the context of the information needs of patients diagnosed with life-threatening conditions, such as advanced cancer [32]. A key finding from this study was that unmet information needs include better understanding of disease trajectory and future care needs, better access to palliative care information, and more participation in medical decision-making. This finding is consistent with previous qualitative research demonstrating that a clear desire for appropriate information was expressed by the majority of cancer patient [33]. Moreover, our study showed that late-stage YMA cancer patients want more information to be able to medical decisions-making in. Medical decision-making is a central component of care for cancer patients, and modern medical practice places great emphasis on the self-determination of individuals to choose what to do or not to do for them [34]. Therefore, when addressing the unmet information needs of YMA patients with malignant tumors, medical professionals should assess the root causes of the patients’ unmet information needs and provide counseling and health literacy training to the patients. Additionally, it should be considered that YMA patients with malignant tumors acquire information in more informative ways, such as browsing professional medical websites, forums, social media, and apps.

Future research should prioritize evaluating the efficacy of telehealth in rural palliative care access, exploring cultural variations in care preferences to improve equity, and assessing long-term outcomes of early palliative interventions in non-cancer populations (e.g., heart failure, dementia). Additionally, studies on scalable models for training primary care providers in low-resource settings and the impact of non-pharmacological therapies (e.g., music, art) warrant further investigation. Policy-focused research could analyze cost-effectiveness of home-based palliative programs to inform healthcare reforms.

This qualitative study was conducted in a cancer hospital which might limit the applicability of its findings. Further, interviews with families and caregivers were not conducted, and the sample size was limited in number. In the future, interviews with families and caregivers can be conducted and the sample size expanded and diversified to make the research results more representative. In addition, only patients with advanced tumors were selected for this study. In the future, we can further explore the different needs of early-stage cancer patients.

## 5. Conclusions

There are multiple issues regarding the implementation of palliative care for YMA patients with malignant tumors, and the identification and fulfillment of palliative care needs require further improvement. The needs for palliative care among YMA patients with malignant tumors mainly include symptom management needs, psychological support needs, social support needs, and information needs. This study will combine these four thematic contents to provide a reference basis for the construction of subsequent scale items.

## Figures and Tables

**Table 1:** Semi-structured interview guide.

Semi-Structured Interview Guide	
1	What do you know about your illness?
2	What treatments have you received since the diagnosis of this disease?
3	What symptoms are currently making you feel uncomfortable? Or are there pressing issues that need to be addressed?
4	Do you know about palliative care? What do you know about palliative care?
5	What do you think of palliative care?
6	Would you choose palliative care at this stage and later? Why?
7	What needs are there in the past or future that might be addressed by palliative care?
8	What other needs do you have for psychological, social, or religious support?
9	If there was a palliative care presentation, what would you be most interested in learning about?
10	Is there anything else you would like to add?

**Table 2:** General data of research subjects (*n* = 16).

No.	Gender	Age (Years)	Education Level	Monthly Household Income (In CNY)	Treatment	Type of Cancer	Cancer Staging	Time to Diagnose
P1	male	54	Junior high school and below	not have	Chemotherapy + Surgery	cardiopulmonary	Phase IV	Less than 12 months
P2	male	47	Junior high school and below	3000–4999	Radiotherapy	cardiopulmonary	Phase IV	Greater than 24 months
P3	male	58	Junior high school and below	3000–4999	Radiotherapy + Chemotherapy	cardiopulmonary	Phase IV	Greater than 24 months
P4	male	56	Junior high school and below	less than 3000	Radiotherapy	cardiopulmonary	Phase IV	Less than 12 months
P5	male	55	Junior high school and below	5000–9999	Radiotherapy	hepatic pancreas	Phase Ill	Greater than 24 months
P6	female	29	College	3000–4999	Radiotherapy + Chemotherapy	oral and maxillofacial	Phase IV	Less than 12 months
P7	male	55	Junior high school and below	3000–4999	Chemotherapy + Surgery	gastrointestinal	Phase IV	12–24 months
P8	female	31	College	3000–4999	Radiotherapy + Chemotherapy + Surgery	cardiopulmonary	Phase IV	Greater than 24 months
P9	female	58	College	5000-9999	Chemotherapy + Surgery	gastrointestinal	Phase IV	12–24 months
P10	male	56	Junior high school and below	3000-4999	Radiotherapy + Chemotherapy	gastrointestinal	Phase IV	Greater than 24 months
P11	male	54	College	10,000 or more	Radiotherapy + Chemotherapy + Surgery	urology	Phase IV	Greater than 24 months
P12	female	38	College	10,000 or more	Radiotherapy	gastrointestinal	Phase IV	12–24 months
P13	male	48	Junior high school and below	5000–9999	Radiotherapy	cardiopulmonary	Phase IV	Less than 12 months
P14	male	27	College	3000–4999	Radiotherapy	oral and maxillofacial	Phase Ill	Less than 12 months
P15	male	24	College	3000-4999	Radiotherapy + Chemotherapy	oral and maxillofacial	Phase IV	12–24 months
P16	male	37	College	10,000 or more	Radiotherapy	oral and maxillofacial	Phase IV	Less than 12 months

## Data Availability

Data cannot be shared publicly because of the confidentiality statement given to the patient. Data are available from the Chongqing Cancer Hospital Institutional Ethics Committee (contact via czll6545@126.com) for researchers who meet the criteria for access to confidential data.
